# *Bacillus* sp. Acting as Dual Role for Corrosion Induction and Corrosion Inhibition with Carbon Steel (CS)

**DOI:** 10.3389/fmicb.2017.02038

**Published:** 2017-10-24

**Authors:** Santosh K. Karn, Guan Fang, Jizhou Duan

**Affiliations:** ^1^Key Laboratory of Marine Environmental Corrosion and Biofouling, Institute of Oceanology, Chinese Academy of Sciences, Qingdao, China; ^2^Department of Biotechnology, National Institute of Technology, Raipur, India; ^3^Department of Biochemistry and Biotechnology, Sardar Bhagwan Singh Post Graduate Institute of Biomedical Science and Research, Dehradun, India

**Keywords:** *Bacillus* sp. corrosion inhibition, corrosion induction, peroxidase, catalsae, biofilms

## Abstract

Present work investigated the role of five different bacteria species as a corrosion inducer as well as corrosion inhibitor with carbon steel (CS). We observed the ability of different bacteria species on the metal surface attachment, biofilm formation, and determined Peroxidase, Catalase enzyme activity in the detached biofilm from the CS surface. We found that each strain has diverse conduct for surface attachment like DS1 3.3, DS2 2.5, DS3 4.3, DS4 4.0, and DS5 4.71 log cfu/cm^2^ and for biofilm 8.3 log cfu/cm^2^. The enzyme Peroxidase, Catalase was found in huge concentration inside the biofilm Peroxidase was maximum for DS4 36.0 U/ml and least for DS3 19.54 U/ml. Whereas, Catalase was highest for DS4, DS5 70.14 U/ml and least 57.2 U/ml for DS2. Scanning electron microscopy (SEM) was conducted to examine the biofilm and electrochemical impedance spectroscopy (EIS) were utilized to observe corrosion in the presence of bacteria. The electrochemical results confirmed that DS1, DS3, DS4, and DS5 strains have statistically significant MIC-factors (Microbially Influenced Corrosion) of 5.46, 8.51, 2.36, and 1.04, while DS2 protective effect factor of 0.89. Weight reduction results with carbon steel likewise supports that corrosion was initiated by DS1 and DS3, while DS2 and DS5 have no any impact though with DS4 we watched less weight reduction however assumed no role in the corrosion. We established the relation of Peroxidase enzyme activity of the isolates. DS1, DS3 and having Peroxidase in the range 22.18, 19.54 U/ml which induce the corrosion whereas DS2 and DS5 having 28.57 and 27.0 U/ml has no any effect and DS4 36 U/ml has inhibitory effect, increasing concentration inhibiting the corrosion. For Catalase DS1, DS3 have 67.28, 61.57 U/ml which induce corrosion while DS2 and DS5 57.71 and 59.14 U/ml also has no effect whereas DS4 70.14 U/ml can inhibit corrosion. Results clearly express that in a specific range both enzymes can induce the corrosion. Our goals are to pursuit and locate the potential role of the enzyme in corrosion induction and inhibition. There is still further work is proceeded for the more profound perception.

## Introduction

Microbes colonize a wide variety of habitats. The main reasons for this success are diversity flexibility in metabolic strategies, ability to change growth patterns and adapt to changes in the growth conditions. Microbiologically influenced corrosion (MIC) is a physical, chemical, and microbiological process in which the dynamic support of microorganisms initiates, facilitates, or accelerates corrosion and electrochemical processes (Beale et al., 2010; Kip and van Veen, 2015). The development of biofilms on objects dipped into a water domain is an aggregate activity. It is brought about by microbial growth and the production of organic and inorganic macromolecules, including exopolymeric substance (EPS), facilitating hydrodynamic erosion. EPS modifies the properties of the substratum surface including surface free energy, surface charge, wettability, and consequential in either induce or slow down the corrosion (Gunasekaran et al., 2004). Biofilms formation on metals begins instantly after metal immersion into the fluid condition, and may be patchy (Characklis and Marshall, 1990). In initial stages, a thin film of organic and inorganic molecules of relatively high molecular mass was created. At this stage, the film would already be able to change the electrostatic charge and immerse/soak on the metal surface, encouraging its further colonization by microorganism. In a brief timeframe relying upon the watery condition in which the metal is submerged, microbial development and creation of different metabolite at that point may bring about the advancement of biofilms.

The dominant organism in connection to biocorrosion are the sulfate reducing bacteria (SRB), However, recent investigation suggest that SRB require not to be available in plenitude in every single microbial group in charge of MIC (Zhu et al., 2003; Jan-Roblero et al., 2004). The dominant microorganism related with structure failures of mild steel, stainless steel (SS), and cast iron structures are sulfur oxidizing bacteria, SRB, manganese oxidizing bacteria, iron-oxidizing/reducing bacteria, and the organism releasing organic acids and exopolymers or slime. All of these can exist together in ordinarily happenings in biofilm, consistently encircling synergistic group (consortia) that are fit for impacting electrochemical processes through obliging metabolism not found in the individual species. Corrosion costs 4% of the GDP of the industrialized nation and the 20% of this cost is evaluated to be because of the activity of microorganisms (Koch et al., 2002).

The impact of enzymes has been recently investigated: Peroxidase and Catalase are of specific worry, as they have been recognized in some marine biofilms. The enzymes and proteins associated possess a heme-group (also called hemins) which is engaged with the transfer of electrons during the enzymatic catalysis. Because microbes derive their energy by oxidizing and reducing a variety chemical compounds from natural resource; they can also change their metabolic activities and release various proteins, sometimes leading to MIC is one of them (Landoulsi et al., 2008). Therefore, in the present investigation microorganisms were isolated from the corroded carbon steel sample that was kept in a sea water for about 2 years and microorganism were isolated used for experimental biofilms formation on carbon steel surfaces during which enzymatic activities were observed in relation to both corrosion stimulation and corrosion inhibition.

## Materials and methods

### Sample collection, bacterial isolation, and purification

Sample was collected from the corroded material Q235 (carbon steel). A fresh inner rust layer was collected from the most damaged site of corroded surface after the outer layer had been scraped from the damaged area of the corroded surface with sterile scalpel from the sea water at the Marine Corrosion Experiment Station near Qingdao, Shandong, China (NL 36°03, EL 120°25), normal seawater temperature 13.7°C, dissolved oxygen 8.4 mg/l, salinity 32 PSU, pH “8.3.” The material was removing after transferred, to a sterile container. Bacterial isolation was done by serial dilution into Winogradsky nutrient medium containing (g/l): 1.27 NaNO_3_; 1.0 K_2_HPO_4_; 0.1 CaCl_2_; 0.2 MgSO_4_.7H_2_O; 0.05 FeSO_4_.7H_2_O; 0.54 NH_4_Cl; and 10 D-glucose anhydrous, supplemented with 0.5 yeast extract (pH 4.8). Morphologically different colonies were isolated and purified on the same medium continuously up to their successive generation. Morphologically five distinct colonies were screened and purified further by repeated subculture on the suitable medium. After initial screening, we observed that five isolated strains having a Fe-oxidizing ability on the Winogradsky medium. These strains are designated as DS1, DS2, DS3, DS4, and DS5. All cultures used in this study were grown under aerobic conditions.

### Identification based on 16srRNA gene sequencing

Genomic DNA was extracted, next amplification of 16SrRNA gene was done by utilizing polymerase chain reaction (PCR; LS-P96G, LabServ-China) from purified genomic DNA by following Karn et al. (2011). The amplified product was further purified with QIA gel extraction kit (Qiagen, Gaithersburg, MD, USA) and amplicons were sequenced using a DNA sequencer [Applied Biosystems (Forster City, CA, USA) 3130 XL Genetic Analyzer; DNA Sequencing Facility, Shanghai Sunny Biotechnology Co. Ltd., China]. Basic Local Alignment Search Tool (BLAST) and ribosomal database (RDB) database II (http://www.ncbi.nlm.nih.gov/) was used analyze the accessible DNA sequence in GeneBank.

### Role of microorganisms in attachment to CS

Attachment of bacteria strains DS1, DS2, DS3, DS4, and DS5 was performed on previously grown stationary phase cultures further diluted in sterile water to obtained cfu/ml about 2 log. Now 50 ml test-tube containing carbon steel coupons was immersed with 20 ml diluted culture, further incubated for attachment for 12 h at 30°C. After that it was removed carefully using forceps and kept in 100 ml sterile double distilled water and smoothly agitated for 15 s at room temperature. Next the coupons was clean using sterile distilled water 200 ml with stirring for 5 s and dipped in 50 ml centrifuge tubes with beads (1 g) encompass with phosphate buffer saline (PBS) 30 ml. The PBS with carbon steel coupons was vortexes at highest rate for 1 min instantly after vortexing; suspensions were serially diluted in sterilized distilled water and transferred to the Windrogasky agar plate to determine the populations of bacteria. The number of bacterial colonies (cfu) was observed after incubation for 24 h at 30°C.

### Role of microorganism in biofilms formation

DS1, DS2, DS3, DS4, and DS5 strains grown-up to unmoving phase in the Winogradsky medium for 24 h at 30°C further centrifuged (6,000 rpm, 10 min, 4°C), and the cells were suspended in saline solution (pH 7.4) to obtain (7 log cfu/ml). Suspension of cell was maintained at 4°C not more than 30 min to prevent the changes physiological activity and number of microorganism. Further 50 ml test tube encompass with carbon steel coupon was deposited with suspensions (20 ml) of each strain and incubated at 4°C for 24 h furthermore it was removed by forceps and kept in 100 ml distilled water for 15 s and washed with sterile water with gentle agitation for 5 s. The washed CS coupons was dipped in 20 ml Winogradsky liquid medium in glass tubes, and then incubated at 30°C for 10 days. Biofilm formed on the CS coupons by each culture was determined after 24 h (dipped in culture at 4°C) 0 days, and after 10th days of incubation. Further coupons were taken out from the suspension and washed for 15s by proper agitation in 200 ml of sterile water. Again another rinse was given for 5 s with agitations in 100 ml sterile water then the coupons was kept in 50 ml centrifuge tube encompass with glass bead (3 g) and PBS buffer (20 ml) and mixed by vortexing for 1 min. The PBS suspension of each culture were diluted serially and plated on agar medium and incubated for 24 h. After that developed colonies on the plated were counted.

### Peroxidase enzyme assay in the biofilms

About 10 mg or 100 μl of detached biofilm of each culture was used to observe the presence of Peroxidase enzyme activity from the surface by spectrophotometer as described by Rajkumar et al. (2013). Absorbance was taken at 414 nm using citrate phosphate buffer (100 mM), ABTS 1.7 mM (2, 2 azinobis 3 ethyl benzo thiazolin 6 sulphonic acid) H_2_O_2_ (2.5 mM; total reaction mixture 1 ml). Furthermore, to analyze 0.1 ml of biofilm, 0.9 ml of ABTS and 25 μl of H_2_O_2_ were taken and the optical density (OD) was read at 414 nm for 1 min. For control a sample containing 0.1 ml of distilled water, 0.9 mL of ABTS and 25 μl of H_2_O_2_ was kept as blank. To measure changes in absorbance heat-denatured enzyme sample served as control. Changes in absorbance of 1.0/ml/min at 414 nm defined as one unit of Peroxidase enzyme. All these steps were performed cooled on ice to prevent loss of the active enzyme.

### Catalase assay in the biofilms

Detached biofilms (10 mg) was taken and suspended in 100 ml of isotonic solution. An assessment of enzymatic activities was determined by Iwase et al. (2013) by plotting a standard curve with the known concentration (or unit of enzyme activity). Standard Catalase was prepared separately in distilled water for each concentration (Catalase from bovine liver 2,950 units/mg solid; 65% protein; 4,540 units/mg protein, the product number C1345, Sigma-Aldrich). Furthermore, bacterial suspension (100 μl) was taken to Pyrex tube (13 mm diameter, 100 mm height, borosilicate glass; Corning, USA). Afterwards, 100 μl of 1% Triton X-100 and 100 μl of undiluted hydrogen peroxide (30%) was taken to the solution and mixed thoroughly and incubated at room temperature. Then the height of the O_2_-forming foam was measured using a ruler. We measured the O_2_-foam which was constant for 15 min. All these steps performed under ice condition (sample was kept in an ice bucket) to prevent the loss of active enzyme.

### Electrochemical experiment

A three-electrode system was used for carrying the electrochemical experiments. Round working electrode with diameter of 0.5 cm was used; the connection was made through copper wire to the back of coupon and sealed with epoxy resin. Platinum electrode and saturated calomel electrodes (SCE) were used as the counter and reference electrode separately. The surface of working electrode was polished sequentially up to P5000 grit SiC papers, followed by cleaned in ethanol.

All the electrodes were put in a sterile environment under ultraviolet light illumination for at least 30 min to ensure no contamination by other bacteria. A control experiment triplicate (without inoculant) was conducted to explore the influence of biofilms on the electrochemical behavior of carbon steel. An electrochemical measurement was performed every-day during 10 days of exposure. Electrochemical impedance spectroscopy (EIS) measurements were made with Solartron 1287/1260 electrochemistry apparatus. The potential amplitude was 10 mV vs. the open circuit potential (OCP) and the frequency range was ranged from 10^5^ to10^2^ Hz. Potentiodynamic polarization curves were obtained from −800 to 1,200 mV vs. *E*_*cor*r_ at a potential scanning rate of 0.5 mV/s. The results were fitted by Corr View-Electrochemistry software to get the electrochemical parameter.

### Weight loss of carbon steel (corrosion rates)

Calculation of corrosion rates was based on the analysis of the weight loss by measuring the sample mass before and after corrosion as shown in Equation (1). All measurements were conducted in a sterile environment using AIRTECH clean chamber after 30 min of ultraviolet light sterilization ensure a sterile environment.

(1) $$\begin{array}{l} \upsilon =\frac{m_{1}-m_{2}}{AT\rho } \end{array}$$

Where υ is corrosion rate of carbon steel in mm y^−1^; m_1_ is mass of coupon before corrosion in g; m^2^ is mass of coupon after corrosion in g; *A* is the surface area of carbon steel in mm^2^; *T* is exposure time in *y*; and ρ is metal density in g mm^−3^.

### Scanning electron microscopy (SEM) analysis

Scanning electron microscopy (SEM) was applied to examine the surface appearance of specimens after the biofilms formation. SEM was used to observe the biofilms from the steel surface by using pretreatment of stainless and carbon steel by standard protocols. Pretreatment of biofilms was done by using 10, 25, 50, 75, and 100% ethanol at different time intervals. Further it was treated with gluteraldehyde and dried properly under vacuum. Then it was used to observe biofilms using an SEM S-3400N (Hitachi, Japan).

### Statistical analysis

All examinations were performed in three duplicates and two specimens for each test parameter were investigated at each examining time. Additionally investigation was performed utilizing GraphPad Prism (variant 4.03) programming (GraphPad, CA, USA). Information was factually examined by analysis of variance (ANOVA) and the mean contrasts were analyzed by Tukey-Kramer numerous correlation test at *p* < 0.05.

## Results and discussion

### Isolation and characterization of microorganisms

Bacterial strains were isolated and after initial screening, we observed that isolated strains having Fe-oxidizing ability grew on the Winogradsky medium. Further, all five strains were characterized by 16S rRNA gene sequencing, the sequences were looked at against the accessible DNA groupings in GeneBank utilizing BlastN scan apparatus from National Center for Biotechnology Information (NCBI). The BlastN analysis shown for DS1, DS2, DS4, and DS5 sequences showed 99% of sequence identity (*E*-*value-0.0*) with *Bacillus licheniformis* whereas DS3 showed close homology to *Bacillus* sp. Further, all five sequences were submitted to GeneBank at NCBI and their accession numbers are DS1 (KM434196); DS2 (KM434197); DS3 (KM454976); DS4 (KM454977); DS5 (KM454978). *B. licheniformis* is pervasive in nature, existing predominately in soil as spores. Basically *B. licheniformis* is facultatively anaerobic; therefore it is able to survive in the inner layer of carbon steel taking into account development in extra environmental specialties. The microorganism is typically saprophytic in nature and its generations of proteases and capacity to separate complex polysaccharides empowers it to contribute generously to supplement cycling (Claus and Berkeley, 1986). Certain individuals from this species are critical in bacterial denitrification in the earth (Alexander, 1977).

### Attachment of microbial strains to carbon steel

Surface attachment of microbial strain DS1, DS2, DS3, DS4, and DS5 to carbon steel was observed after 12 h. The number bacterial cells attached to the surface of CS are shown in Table 1. Strain DS3 and DS5 attached, significantly more to the carbon steel surface 4.3 and 4.7 cfu/cm^2^ than other microbes. But in planktonic cell or liquid culture we found highest cfu/ml values for DS5, followed by DS4, DS3, DS1, and DS2, respectively, for carbon steel. Early stationary phase culture was selected for the present investigation because to minimize the potential influence of different physiological states on attachment and biofilms formation. Other researcher like (Hood and Zottola, 1997) have determined that nutrients and other components in media affect attachment of microorganisms to surfaces of various materials. Hood and Zottola (1997) concluded that composition of growth and conditioning media influence the attachment of *Salmonella enterica, Serovar typhimurium*, and *Listeria monocytogenes* to SS surfaces. Surface attachment and biofilm formation on metal surfaces is closely associated with MIC. Microorganisms attach and produce specific chemistries that are corrosive and specific to environments that produce electrochemical reactions, and these conditions lead to corrosion. The microorganism when confronted with a given selective situation; it follows a similar evolutionary path. However, once the selection pressure is resumed, the organism is tends to be out-competed by non-evolved partners. Several research groups worked on unraveling MIC mechanisms; and found that bacterial metabolism may produce aggressive substances such as, oxidants, reductants, acids, and complexing agents. This may lead to local patches of low pH and redox potential beneath the cluster of biofilms and promote corrosion by forming complexes and thus solubilizing the metals by complex formation (Dowling and Guezennec, 1997).

**Table 1:** Number of attached bacterial cells with carbon steel surface and bacteria present the culture fluids determined in log cfu/cm^2^ and log cfu/ml.

**Serial no**.	**Isolates**	**On CS surface**	**In culture fluid**
1	DS1	3.3 ± 0.2	4.7 ± 0.3
2	DS2	2.5 ± 0.3	4.6 ± 0.2
3	DS3	4.3 ± 0.21	5.3 ± 0.2
4	DS4	4.0 ± 0.1	6.1 ± 0.2
5	DS5	4.71 ± 0.2	7.1 ± 0.31

### Formation of biofilms

Formation of biofilms was observed in all these isolates on carbon steel coupons after surface attachment observation. We found that all the strains formed easily observable biofilms on the coupons. The number of colonies formed on the coupons was observed at 0 days and after 10 days. At 0 days with carbon steel coupons, DS4 had the highest number 1.9 cfu/cm^2^ of colonies DS1and DS3 showed significantly high numbers of colonies 1.4 and 1.6 cfu/cm^2^. DS2 and D5 showed only 1.0 cfu/cm^2^ and after 10 days colonies sowed about 8.8 cfu/cm^2^, which demonstrate the biofim forming ability of the present strain. DS1, DS2, DS4 all formed about 7.8 cfu/cm^2^ of colonies on CS coupons. The results from the coupons showed that all five strains produced colonies. Details of the results in details are presented in Table 2. Biofilm formation allowed these first sessile organisms to remain in place, and to trap and utilize the scarce organic compounds. Importantly the factor regulating attachments of bacteria onto surfaces is nutrient availability, liquid flow, and the electrochemical properties. At the point when supplements are non-constraining in the fluid stage there is no requirement for the microscopic organisms to append themselves. Stress circumstances like an exhaustion of supplements makes sessile development better in streaming fluids (O'Toole et al., 2000).

**Table 2:** Number of cell/colonies in biofilm on the carbon steel surface before after treatment (1st and 10th days) represented in term of (log cfu/cm^2^).

**Serial no**.	**Isolates**	**At 1st days**	**At 10th days**
1	DS1	1.4 ± 0.1	8.15 ± 0.3
2	DS2	1.05 ± 0.5	7.85 ± 0.3
3	DS3	1.6 ± 0.1	8.8 ± 0.32
4	DS4	1.9 ± 0.2	8.4 ± 0.24
5	DS5	1.1 ± 0.1	8.8 ± 0.25

### Peroxidase enzyme analysis

Biofilms detached from the coupons were assayed for Peroxidase and in culture fluid/supernatant to observe the proportion of this enzyme. We found highest Peroxidase enzyme with DS4 ~36 U/ml and least with DS3 ~19.54 U/ml with biofilms and almost no Peroxidase was observed in control detail mentioned in Table 3. Landoulsi et al. (2008) confirms that role of enzyme may be in the depolarization of oxygen reduction reaction on SS. Furthermore, Peroxidase was also observed in the culture fluid in which coupon was dipped for each culture with carbon steel. With carbon steel we found about 6–7 U/ml except DS4 has a little less concentration about 4 U/ml. By observing the result of Peroxidase we found that DS1 and DS3 having 22 and 19.54 U/ml of enzyme and played a role in corrosion behavior whereas DS2 and DS5 about 27 and 28 U/ml and DS4 highest concentration. We evaluated the Peroxidase activity with the concentration of microbes we observed enzymatic activity depend on the response of the microbes to the coupons and their metabolic activity and expression of enzyme. In the present observation, we found that increased concentration of enzyme has an inhibitory effect on corrosion after increasing a certain limit these enzymes can inhibit the corrosion. Current results is an agreement of finding by Imanaka et al. (1983) observed that the difference in corrosion causing capacity among different bacterial was most probably caused by the differences in enzymes expression within the biofilms. Recently more concentration has been paid on the enzyme Peroxidases since they assume significant part in the corrosion process. At the same time biofilms also generate Catalase for overcoming the harmful nature of hydrogen peroxide. Landoulsi et al. (2008) suggested about the oxygen utilization through metabolic pathways in the biofilms impacts its accessibility of the metal/biofilms interface. Thus, oxygen inclinations result from its dispersion in the biofilms and from its utilization in metabolic pathways too. In microorganisms, oxygen goes about as a last electron acceptor. The significant piece of oxygen experiences a four-electron-pathway reduction in microorganisms, as indicated by the response. This response is catalyzed by cytochrome-c oxidase.

12O2+2H++2e−→H2O

In the course of the respiratory procedure, oxygen is devoured by *Bacillus* sp. furthermore, *Serretia* sp. (a naphthalene debasing strain) and changed over into water, wherein H^+^ is utilized from the corrupted create and electrons are provided by a cytochrome oxidase catalyst (Muthukumar et al., 2003; Rahman et al., 2003).

**Table 3:** Peroxidase and catalase concentrations observed in the detached biofilm and in supernatant from carbon steel Unit/ml.

**Serial no**.	**Isolates**	**Peroxidase**		**Catalase**	
		**Biofilm CS**	**Culture fluid/Supernatant**	**Biofilm CS**	**Culture fluid/Supernatant**
1	DS1	22.18 ± 3	7.14 ± 2	67.28 ± 2	5.85 ± 2
2	DS2	28.57 ± 1	7.8 ± 2	57.71 ± 3	7.28 ± 1
3	DS3	19.54 ± 1	6.3 ± 3	61.57 ± 2	5.85 ± 2
4	DS4	36.0 ± 2	4.1 ± 1	70.14 ± 4	8.71 ± 2
5	DS5	27.0 ± 3	6.7 ± 1	59.14 ± 2	8.42 ± 1
6	DS0	0.32 ± 0	0 ± 0	13 ± 1	0 ± 0

*DS0 is treated as the control*.

### Catalase enzyme analysis

Catalase was observed in the detached biofilms and culture fluid is described in the (Table 3). DS4 has shown maximum activity ~70.14 U/ml with carbon steel and potentially no effect on corrosion. Whereas, DS1 and DS3 have shown ~67 and 61 U/ml. DS2 has ~57 U/ml and DS5 has 59.14. Catalase results are comparable with Peroxidase assay and suggesting that the strain can induce and inhibit the corrosion as per the enzyme concentration available within the biofilms. We found Catalase activity also in culture fluid or supernatant ~5 to 9 U/ml. Catalase is a universal cell reinforcement catalyst that degrades hydrogen peroxide into water and oxygen. Previously different hypothesis and literature were reported related to the involvement of some enzyme in the corrosion. Here, we observed the involvement and understand the possible role of Peroxidase, Catalase in aerobic corrosion. Conclusively say that the Peroxidase and Catalase were involved in the corrosion and the bacterial strains that cause the most MIC also produced the highest concentration of these enzymes. Several environmental factors affect the corrosion rate for example metal sorts, substrate synthesis, the mass water, electrochemistry, and microorganisms (Borenstein, 1994). The exact mechanisms of the enzymes in the corrosion process is unclear, but the current study showed that bacterial strains that form biofilm and produce high concentrations of corrosive enzymes within biofilms may play a major role both in the induction and inhibition of corrosion. The systems engaged with MIC are exceptionally entangled, as the procedure is influenced by many variables, including arrangement of differential air circulation cells caused by oxygen breath, generation of corrosive specialists, for example, sulfide by SRB and natural as well as inorganic acids, metal-testimony, hydrogen embrittlement, and metal-restricting impact of extracellular polymeric substance, inactivation of corrosion inhibitors and cathodic and anodic depolarization (Geesey, 1990; Little, 1998). Previously Juzeliunas et al. (2006) observed EPS produced by SRB, having 84–92% proteins and only 8–16% polysaccharides, which increment in the erosion of gentle steel coupons. Which support the presence of enzyme within the biofilm has prospective role in the corrosion. Similarly Morikawa (2006) watched that reductions in consumption rate of aluminum by *Bacillus* sp. biofilms however *Bacillus* sp. which diminished in corrosion rate of aluminum, could increment in corrosion rate when colonized with zinc, and detachment while colonizing gentle steel (Juzeliunas et al., 2006). These outcomes additionally demonstrated that MIC not just relies upon natural conditions and the kind of microorganism, yet additionally on the many variables.

### Electrochemical studies

**Figure 1:**
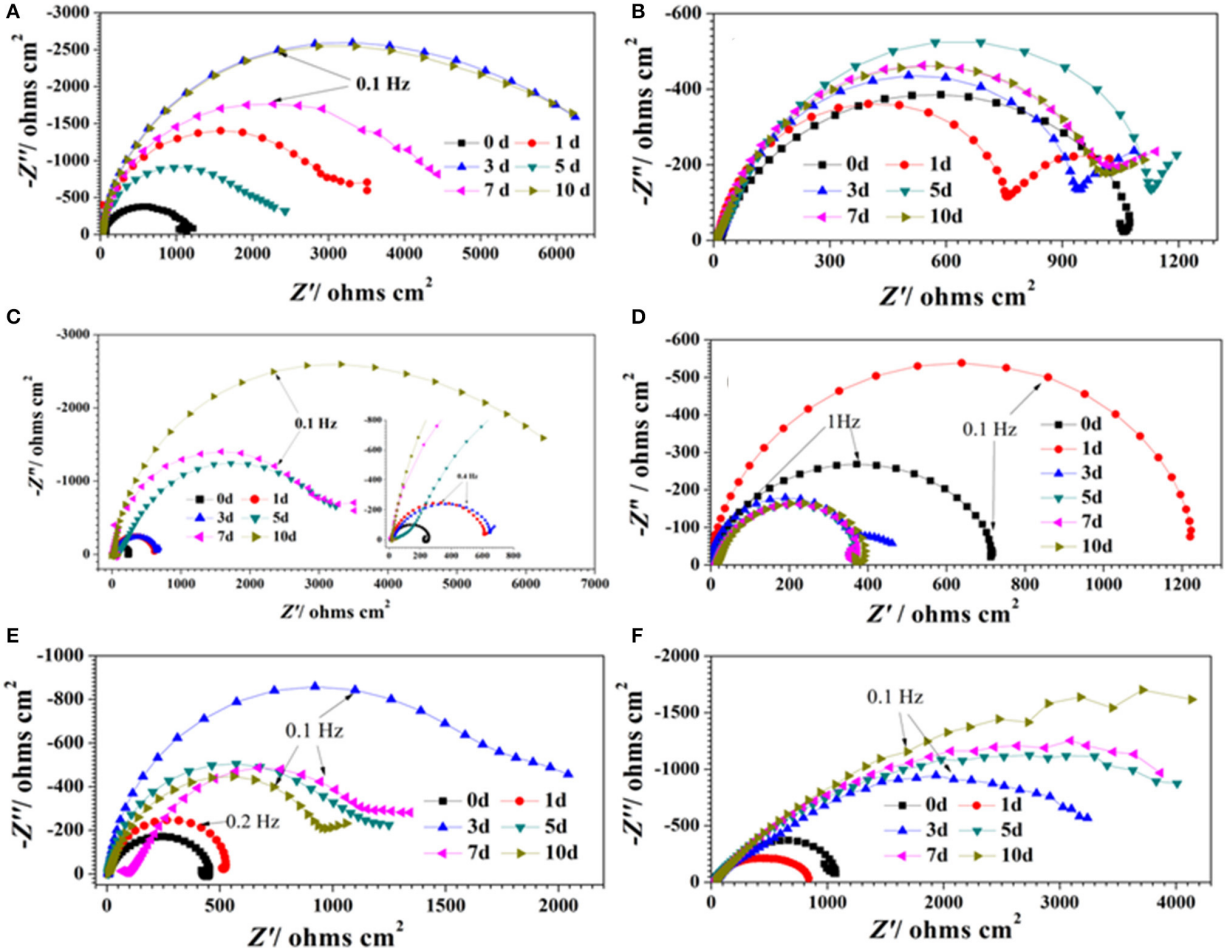
Nyquist plots of carbon steel in different corrosion conditions with time: **(A)** sterile medium; **(B)** DS1 culture; **(C)** DS2 culture; **(D)** DS3 culture; **(E)** DS4 culture; **(F)** DS5 culture.

**Figure 2:**
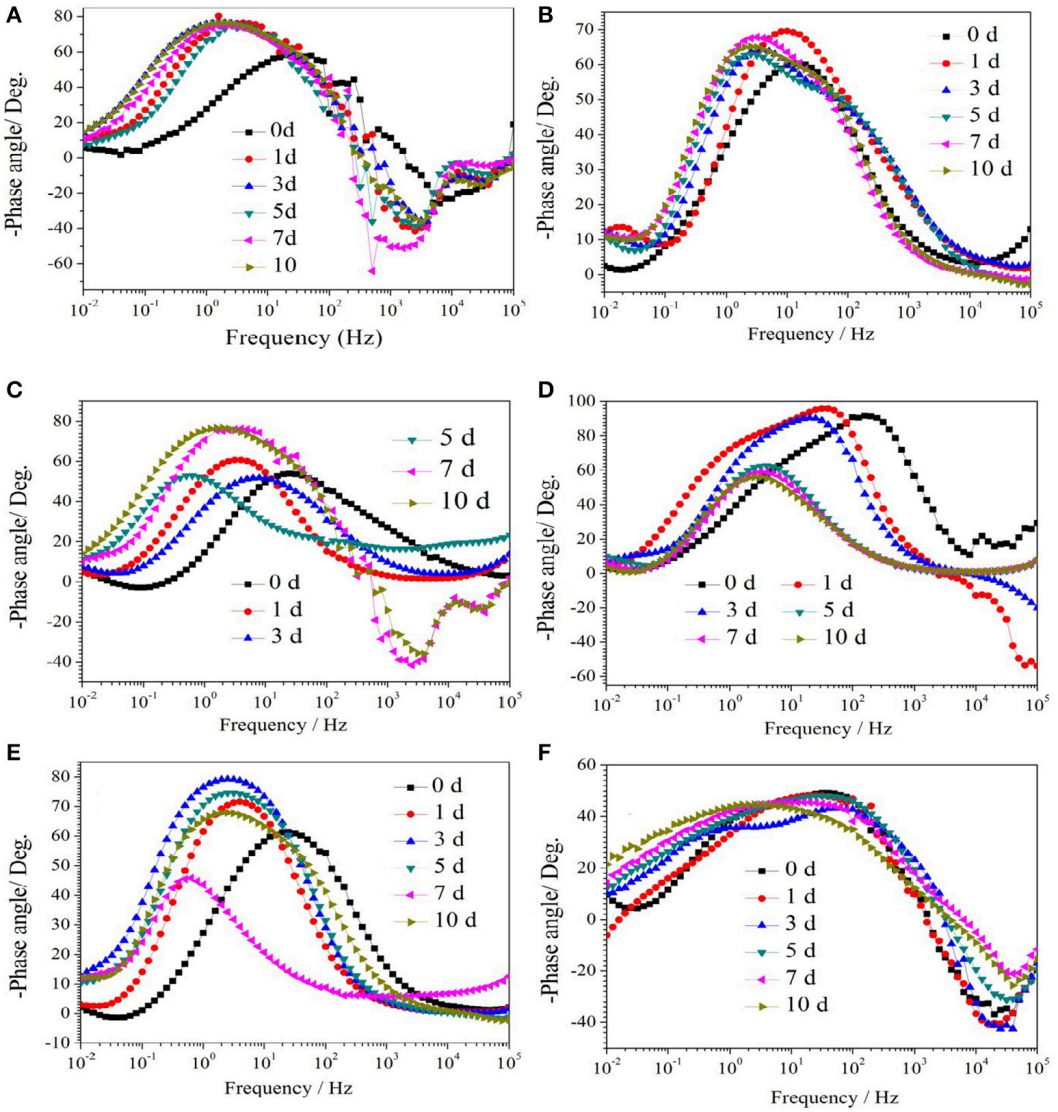
The change of phase angle of carbon steel in different corrosion conditions with time: **(A)** sterile medium; **(B)** DS1 culture; **(C)** DS2 culture; **(D)** DS3 culture; **(E)** DS4 culture; **(F)** DS5 culture.

**Figure 3:**
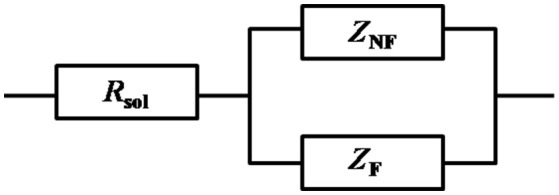
Circuit model of the electrode during the EIS measurement, with solution resistance (Rsol), faradaic impendence (ZF), and non-faradaic impendence (ZNF).

Figure 1 show the Nyquist plots obtained for carbon steel in different environment and the variations of phase angle were shown in Figure 2. The impendence of the steel in different environments had a great difference with each other. The impendence of steel in sterile medium increased and fluctuated as immersion time prolonged. The fluctuation was most probably caused by the corrosion product, and the increase indicated that steel got a restrained corrosion rate in sterile culture medium. The negative value of phase angle in high frequencies maybe associated with the disturbance from the system. However, the impendence of steel in DS1 culture was much lower during the immersion time compared with that in sterile medium. A capacitive semicircle was found at high frequencies, which may be caused by the surface layer due to biofilm formation and the precipitation of corrosion product. At low frequencies, a small capacitive loop was noticed, and this could be attributed to the evolution of the metallic dissolution controlled by charge transfer processes. The peak in phase angle plot Figure 2 was probably caused by charged transfer process. The impendence of steel in DS2 culture medium increased with time which indicated that this bacterial strain inhibited the corrosion behavior of steel during the immersion time. For the DS3 culture, the impendence in Nyquist plot increased on the first day, and then decreased, the magnified Nyquist plot showed that there was a small inductive semicircle at low frequencies from the fifth day, which may be associated with the development of pits. The weight loss data also showed that DS3 caused severe corrosion on the carbon steel. The increase in impendence on the first 3 days in the DS4 culture was probably caused by the biofilms formation, which inhibited the corrosive ions from steel, then the impendence decreased and remained stable, indicating the accelerated corrosion rate of steel. The impendence of DS5 culture increased with immersion time prolonged except the temporary decrease on the first day, the impendence was much lower compared with sterile medium, indicating the higher corrosion rate in DS5 culture. The fluctuation in high frequencies was probably caused by the unstable conditions in the system. While periodic sinusoidal voltage signals are imposed to the working electrode during the EIS measurement, both the faradaic processes (periodical charging and discharging process of double electrode layer) and non-faradaic process (the electrode reaction) happened on electrode surfaces. Therefore, the EIS was the result of a solution resistance (*R*_sol_) and a parallel circuit of faradaic impendence (*Z*_*F*_) and non-faradaic impendence (*Z*_*NF*_), which can be expressed as follows (Figure 3)

(2) $$\begin{array}{l} Z=R_{s}+\frac{1}{\frac{1}{Z_{NF}}+\frac{1}{Z_{F}}} \end{array}$$

At high frequencies, as the *Z*_*NF*_ tend to 0, so the *Z*_f→∞_ was equal to *R*_s_ (Cao and Zhang, 2002; Wu et al., 2014).

At low frequencies, as the *Z*_*NF*_ tend to +∞, the value of *Z*_f→0_ can be expressed as follows:

(3) $$\begin{array}{l} Z_{\text{f}\to 0}=R_{s}+Z_{F}=R_{s}+{Z_{F}}_{\left(\text{f}\to 0\right)} \end{array}$$

Moreover, the polarization resistance (*R*_*p*_) was defined as the faradic resistance when the frequency was 0 (Cao, 2008; Wu et al., 2014).

(4) $$\begin{array}{l} R_{p}={\left(Z_{F}\right)}_{\omega =0} \end{array}$$

During this experiment, the lowest frequency was 0.01 Hz, we can get the value of *R*_*p*_ approximately by the following equation:

(5) $$\begin{array}{l} Z_{p}=|Z_{0,01Hz}|-|Z_{100KHz}| \end{array}$$

Then the corrosion current density (mA/cm^2^) can be obtained as follows:

(6) $$\begin{array}{l} i_{corr}{=}^{B}{/}_{Z_{p}} \end{array}$$

Where, *B* (mV) is an empirical constant and is around at 26 mV for carbon steel in this experiment (Cao and Zhang, 2002).

**Figure 4:**
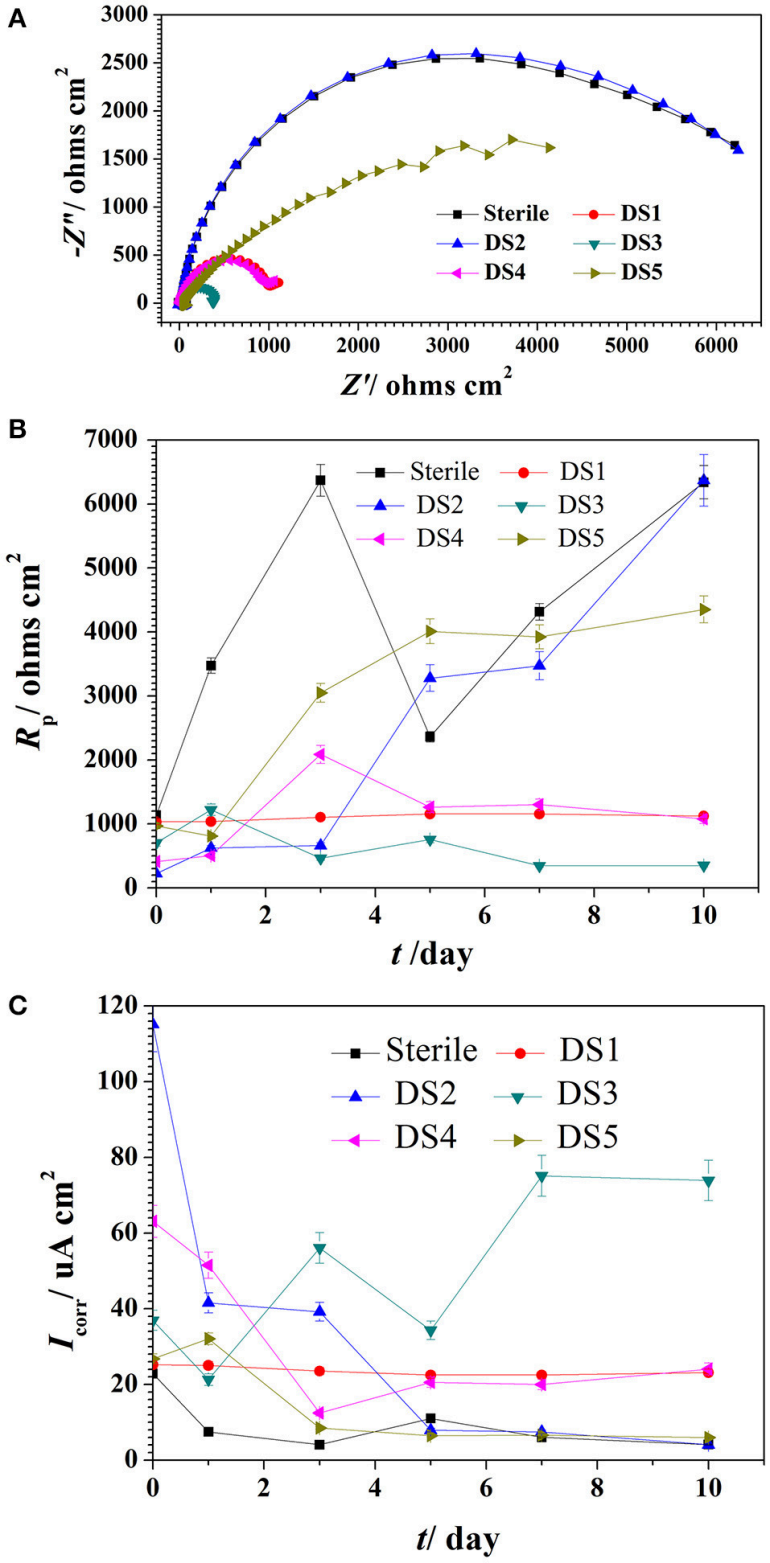
The impendence **(A)**, *R*_*p*_ **(B)**, and *I*_*corr*_ **(C)** of carbon steel in different corrosion conditions.

The Nyquist plot of carbon steel in different corrosion conditions on the 10th day was shown in Figure 4A the impendence of sterile and DS2 culture medium was similar, followed by the DS5, indicating the non-corrosive ability of DS2 and slight corrosive character of DS5. While that of DS3 has the smallest impendence, indicating that carbon steel has the highest corrosion rate in DS3 medium, which was in consistent with the results shown in Figures 4B,C, where steel has the lowest *R*_*p*_ and highest *i*_*corr*_ in DS3 medium. The value of *R*_*p*_ and *i*_*corr*_ fluctuated in the first 5 days and then stayed stable, with the highest *R*_*p*_ and lowest *i*_*corr*_ occurred in sterile and DS2 culture medium, followed by DS5, and then DS1 and DS4 culture medium.

**Figure 5:**
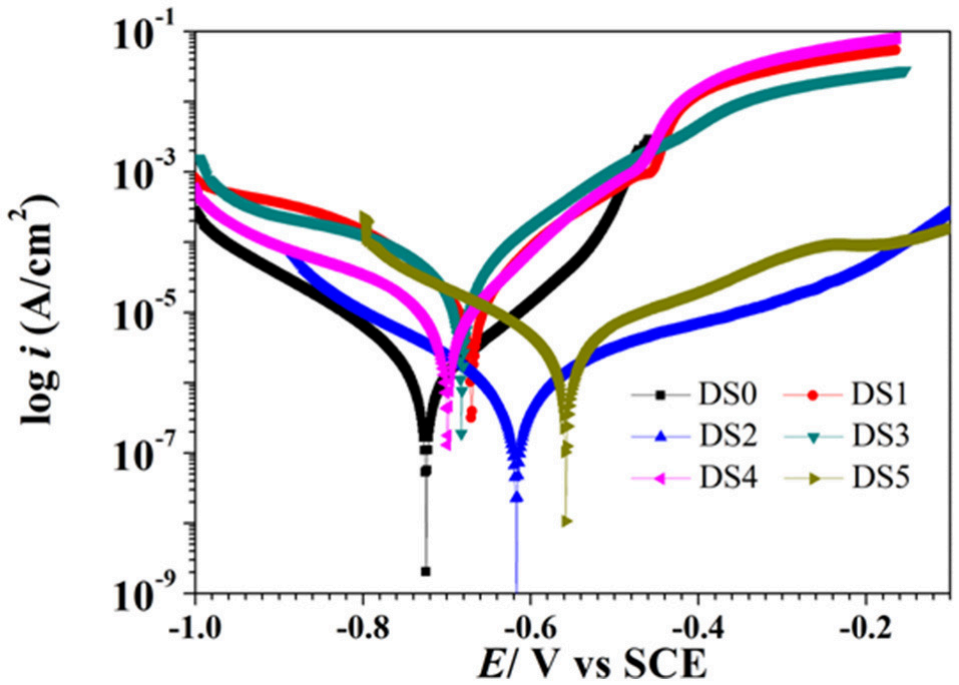
Polarization curves of carbon steel exposed to different corrosion condition on the 10th day.

The potentiodynamic polarization behavior of carbon steel in different medium is shown in Figure 5. The Tafel parameter including cathodic and anodic Tafel slopes (*b*_*a*_,*b*_*c*_),corrosion potential (*E*_*corr*_), as well as corrosion current density (*I*_*corr*_) were obtained from the potentiodynamic polarization curves, and are given in Table 4. By observing the results we found that DS1, DS3, DS4 increased corrosion due to microbial colonization and to the production of a thin film on the metal surface, while DS2 and DS5 had a slight influence on the corrosion rate of carbon steel. Compared with sterile solution, *E*_*corr*_ of carbon steel shifted more toward the positive direction with bacteria; the ennoblement shift was most probably related with the interaction between microorganisms and steel. The electrochemical results confirmed that DS3, DS1, DS4, and DS5 strains showed statistically significant MIC factors of 8.51, 5.46, 2.36, and 1.04,while DS2 protective effect factor of 0.89. Early research conducted by Valencia-Cantero et al. (2003) with two isolates G9a and G9c both strain were taxonomically related to *B. licheniformis*, however, revealed statistically significant MIC factors of 0.54 (a protective effect) and 1.65 (accelerating corrosion effect), respectively. Xu et al. (2013) observed that *B. licheniformis* (ATCC 14580) strain was found create a largest pit depth of 14.5 μm and weight loss 0.89 mg/cm^2^ against carbon steel in 1-week laboratory experiment (accelerating corrosion). Therefore, we find that each strain belong to the same genus behave significantly different with the metal.

**Table 4:** Tafel parameters of the carbon steel exposed to different corrosion condition.

**Condition**	***I*_corr_ (uA cm^−2^)**	***E*_corr_(mV vs. SCE)**	***b*_a_(mVdec^−1^)**	***b*_c_(mVdec^−1^)**	***R*_p_ (ohms cm^2^)**
Sterile	3.433 ± 0.14	−724.7 ± 0.8	131.1 ± 7.6	116.2 ± 12.9	6339.2 ± 260
DS1	18.754 ± 0.23	−671.0 ± 6.3	7108.2 ± 10.7	144.5 ± 4.09	1123.8 ± 45
DS2	3.052 ± 0.07	−683.6 ± 7.4	114.83 ± 11.1	188.1 ± 1.70	6368.4 ± 103
DS3	29.231 ± 0.01	−687.8 ± 6.3	117.06 ± 2.1	116.0 ± 4.41	351.9 ± 25
DS4	8.091 ± 0.22	−699.9 ± 1.5	99.63 ± 4.81	167.7 ± 12.0	1079.6 ± 72
DS5	3.578 ± 0.08	−559.1 ± 0.9	237.1 ± 2.51	153.4 ± 23.18	4351.9 ± 209

### Weight loss

**Figure 6:**
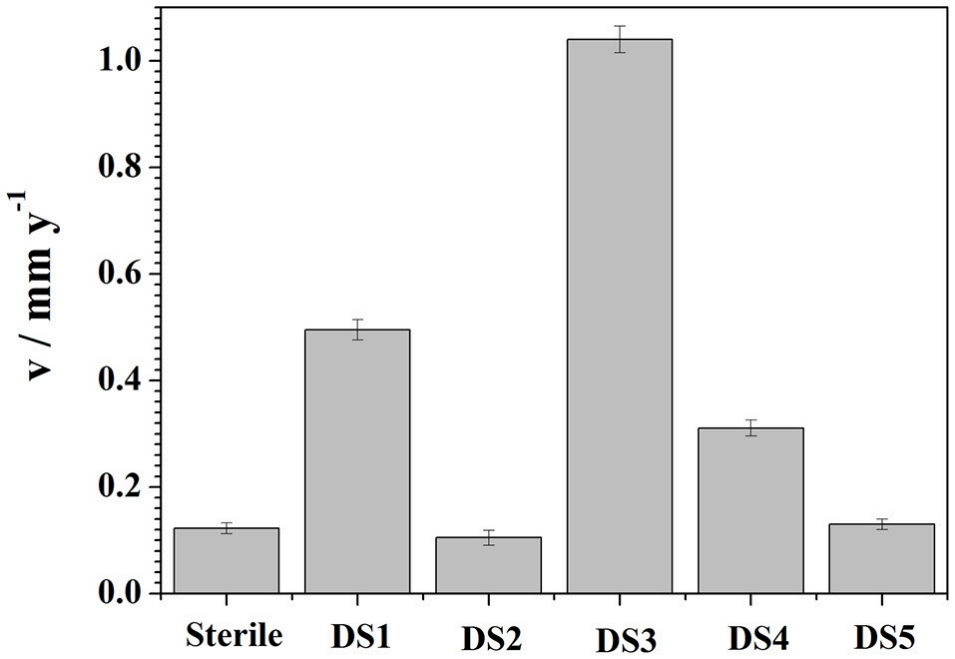
Weight loss results curves of carbon steel exposed to different corrosion condition for 10 days.

Weight loss observation by these isolates with CS was observed and represented in Figure 6. To demonstrate the electrochemical behavior of carbon steel induced by the five bacteria strains, average corrosion weight loss rates of specimens are calculated by the weight loss test. Figure 6 shows the corrosion rates of the specimens after immersing in different bacteria strains for 10 days. The average corrosion rate of the coupons in sterile medium 0.1225 mm y^−1^ (Figure 6), and the corrosion rate had slightly decreased and increased in DS2 and DS5 strains separately. However, the average corrosion rate (1.0401 mm y^−1^) increases significantly in DS3 solution, followed by the DS1 (0.4953 mm y^−1^) and DS4 (0.3106 mm y^−1^) solution. The weight loss results were in consistent with the electrochemical test results. All the results revealed the different microbiological influenced corrosion factors of the five bacteria. However, the electrochemical and weight loss experiment results showed much difference in the corrosion accelerating capacity, with DS1 and DS3 strains acting as a dual role for corrosion induction. While, DS2 and DS5 strains having hardly any impact on corrosion rate changes of carbon steel. The results indicating that bacterium could have different proteins and metabolic activity in accelerating steel corrosion. Zuo (2007) demonstrated that formation of a by *B. licheniformis* biofilm act as a protective layer of corrosion inhibitors on metal surface and thus decreased contact of corrosive agents with metal surface. The study of Örnek et al. (2002) revealed that the *B. licheniformis* could produce γ-polyglutamate, and the secretion of γ-polyglutamate decreased the corrosion rate by 90%, by comparing with *B. subtili*s biofilms which did not secrete polyaspartate or γ-polyglutamate. Previously (Yan et al., 2003; Ubong et al., 2016) was also found that *B. licheniformis* EI-34-6 produced antimicrobial compounds while grown on semipermeable membrane, and inhibited corrosion of steel due to the adhesion of relatively compact and dense biofilms. Studies (Korenblum et al., 2005; Gana et al., 2011) have shown that *Bacillus* could produce antimicrobial peptides, acting as corrosion protectors in biofilms, indicating the potential of *Bacillus* sp. strain as biocontrol agent in corrosion control caused by sulfate-reducing bacteria (SRB) in marine construction. Further studies are needed to clarify the interaction between bacterium and carbon steel considering the difference in corrosion behavior.

### SEM analysis of biofilms

**Figure 7:**
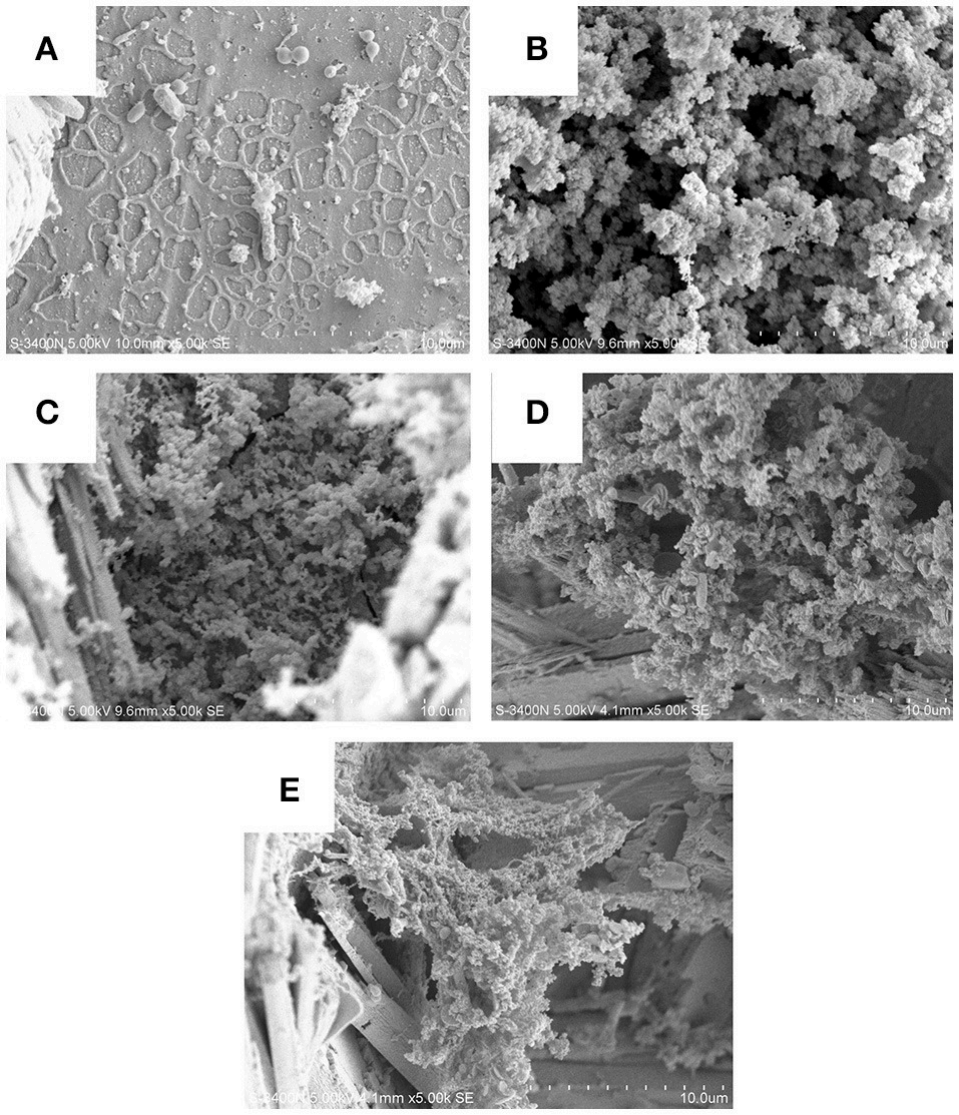
Biofilms and corrosion product on the carbon steel surfaces by producing various metabolites which clearly visible given in the picture of each isolates **(A–E**: DS1, DS2, DS3, DS4, and DS5).

By observing the biofilms we can clearly state that present isolates effectively attached and formed biofilms and bioproduct/corrosion product on the carbon steel surfaces by producing various metabolites which were clearly visible given separate picture of each isolates (Figure 7). On the basis of previously observed outcome with mild steel in *Bacillus thuringiensis* different hydrated species of iron oxides, like Fe_2_O_3_, FeO(OH), Fe(OH)_2_, were found (Hussain et al., 2013). Current data reflect or visualizing the presence of various iron oxide formed in the corrosion products, indicating the role of microorganism the of carbon steel accelerating the microbial corrosion directly to carbon steel.

## Conclusion

Present isolates have high potential for surface attachment it was about 4.0 cfu/cm^2^ and for biofilms formation was about 8.1 cfu/cm^2^ further, we observed Peroxidase and Catalae activity in both biofilms and culture fluid and found maximum Peroxidase for DS4 36.0 U/ml and minimum for DS3 19.54 U/ml, a Catalase maximum for DS4, DS5 70.14 U/ml and minimum 57.2 U/ml for DS2 observed within the biofilm of carbon steel surface. We conclude that at certain range this enzyme can induces the corrosion whereas higher concentrations of both the enzymes can inhibit the corrosion, results support that DS1 and DS3 induce the corrosion DS4 and DS5 has no any effect on corrosion whereas DS2 inhibiting corrosion. Weight loss result also support DS1 and DS3 induces corrosion, whereas DS2 and DS5 have no any effect with DS4 we observed very less weight loss but played no role in the corrosion. Current finding is upheld from the consequences of weight reduction, EIS results. Current finding is supported from the results of weight loss, EIS results. The exact mechanism and enzymatic role is unknown therefore further study is continued to understand the potential role of enzyme.

## Author contributions

SK had created basic research outline, taken laboratory experiments, analysis as well as writing the manuscript. GF helped in conducting experiment and analysis writing of ECE. JD contributed in collection the sample, managed the facilities of experiments, suggested and discussed about the experiments. Moreover, all the authors had gone through the manuscript and revised it technically before submission.

### Conflict of interest statement

The authors declare that the research was conducted in the absence of any commercial or financial relationships that could be construed as a potential conflict of interest.
